# Osteopontin and LDLR Are Upregulated in Hearts of Sudden Cardiac Death Victims With Heart Failure With Preserved Ejection Fraction and Diabetes Mellitus

**DOI:** 10.3389/fcvm.2020.610282

**Published:** 2020-11-30

**Authors:** Mausam Patel, Daniela Rodriguez, Keyvan Yousefi, Krista John-Williams, Armando J. Mendez, Ronald B. Goldberg, Anastasios Lymperopoulos, Leonardo J. Tamariz, Jeffrey J. Goldberger, Robert J. Myerburg, Juhani Junttila, Lina A. Shehadeh

**Affiliations:** ^1^Division of Cardiology, Department of Medicine, University of Miami Leonard M. Miller School of Medicine, Miami, FL, United States; ^2^Interdisciplinary Stem Cell Institute, University of Miami Leonard M. Miller School of Medicine, Miami, FL, United States; ^3^Department of Molecular and Cellular Pharmacology, University of Miami Leonard M. Miller School of Medicine, Miami, FL, United States; ^4^Division of Endocrinolgy, Diabetes and Metabolism, Department of Medicine, The Diabetes Research Institute, University of Miami Leonard M. Miller School of Medicine, Miami, FL, United States; ^5^Department of Pharmaceutical Sciences, College of Pharmacy, Nova Southeastern University, Fort Lauderdale, FL, United States; ^6^Miami VA Healthcare System, University of Miami Leonard M. Miller School of Medicine, Miami, FL, United States; ^7^American Heart Association, Dallas, TX, United States; ^8^Medical Research Center Oulu, Oulu University Hospital, University of Oulu, Oulu, Finland; ^9^Peggy and Harold Katz Family Drug Discovery Center, University of Miami Leonard M. Miller School of Medicine, Miami, FL, United States

**Keywords:** sudden cardiac death (SCD), diabetes mellilus, osteopontin (OPN), LDLR, HFpEF—heart failure with preserved ejection fraction

## Abstract

**Background:** Diabetes mellitus (DM) is associated with increased risk of sudden cardiac death (SCD), particularly in patients with heart failure with preserved ejection fraction (HFpEF). However, there are no known biomarkers in the population with DM and HFpEF to predict SCD risk.

**Objectives:** This study was designed to test the hypothesis that osteopontin (OPN) and some proteins previously correlated with OPN, low-density lipoprotein receptor (LDLR), dynamin 2 (DNM2), fibronectin-1 (FN1), and 2-oxoglutarate dehydrogenase-like (OGDHL), are potential risk markers for SCD, and may reflect modifiable molecular pathways in patients with DM and HFpEF.

**Methods:** Heart tissues were obtained at autopsy from 9 SCD victims with DM and HFpEF and 10 age and gender-matched accidental death control subjects from a Finnish SCD registry and analyzed for the expression of OPN and correlated proteins, including LDLR, DNM2, FN1, and OGDHL by immunohistochemistry.

**Results:** We observed a significant upregulation in the expression of OPN, LDLR, and FN1, and a marked downregulation of DNM2 in heart tissues of SCD victims with DM and HFpEF as compared to control subjects (*p* < 0.01).

**Conclusions:** The dysregulated protein expression of OPN, LDLR, FN1, and DNM2 in patients with DM and HFpEF who experienced SCD provides novel potential modifiable molecular pathways that may be implicated in the pathogenesis of SCD in these patients. Since secreted OPN and soluble LDLR can be measured in plasma, these results support the value of further prospective studies to assess the predictive value of these plasma biomarkers and to determine whether tuning expression levels of OPN and LDLR alters SCD risk in patients with DM and HFpEF.

## Introduction

Heart failure with preserved ejection fraction (HFpEF) is the most common form of heart failure, affecting more than 3 million adults in the United States, and is represented by multiple subgroups or phenotypes (1). Type 2 diabetes mellitus (DM) is present in up to 45% of patients with HFpEF, and is associated with higher rates of morbidity and long-term mortality (2). Pathophysiology of HFpEF with DM include endothelial dysfunction, increased interstitial and perivascular fibrosis, cardiomyocyte stiffness, and left ventricular hypertrophy (3). DM is also associated with an increased risk of sudden cardiac death (SCD), in part linked with the presence of microvascular disease and autonomic neuropathy.

A common cause of non-ischemic SCD in younger subjects is primary myocardial fibrosis (4, 5), which is defined as myocardial fibrosis in the absence of other associated causes of fibrosis, and also has been demonstrated to be a common phenotype in SCD associated with HFpEF and DM (6). Risk stratification within the patient population diagnosed with DM and HFpEF remains a challenge.

Osteopontin (OPN) is a matricellular pro-fibrotic phosphorylated glycoprotein whose upregulation contributes to various pathological diseases such as cancers, chronic kidney disease, atherosclerosis, and adverse cardiac remodeling (7). In experimental models, lower OPN was associated with reduced adipose tissue inflammation, improved glucose tolerance and reduced insulin resistance. We recently showed that genetic knock down of OPN reduced HFpEF pathology in a cardio-renal model of HFpEF in mice (8, 9). Of great significance was the finding that pharmacological blockade of OPN by an OPN aptamer reversed pressure overload-induced heart failure (10).

In HF patients at high risk for SCD, plasma OPN and Galectin-3 levels were associated with sustained ventricular tachycardia and ventricular fibrillation (11). Plasma levels of OPN are increased in patients with heart failure with preserved ejection fraction (HFpEF) and predict mortality (12, 13).

In this study, we investigated the expression levels of OPN and correlated proteins, dynamin2 (DNM2), low density lipoprotein (LDLR), 2-oxoglutarate dehydrogenase-like (OGDHL), and fibronectin-1 (FN1), in the hearts of SCD victims who had both DM and HFpEF. The correlation of the selected proteins with OPN is based on our previous findings that these proteins were positively or negatively correlated with OPN expression in the heart and/or kidney in a mouse model of HFpeF (8, 14). Our hypothesis is that OPN and FN1 may contribute to the extracellular matrix remodeling in HFpEF that can be exacerbated through advanced glycation end products associated with diabetes (15). and may be a target for drug therapy. We reasoned that potential differential expression in the heart, specifically in proteins detected in the plasma such as OPN and LDLR, may identify biomarkers for SCD in patients with DM and HFpEF and generate novel future targets for prevention of SCD.

## Methods

### Sample Collection

A death was classified as sudden if it was either a witnessed event within 6 h of the onset of symptoms or an unwitnessed death within 24 h when the subject was last seen alive in a normal state of health. The current criteria for SCD were chosen to cover as many subjects with SCD as possible, considering that many subjects with SCD would be found dead and would have been missed with the 1 h definition for SCD originally proposed by Hinkle and Thaler (16). All of the subjects are from a defined geographical area in Northern Finland.

Autopsied human heart samples from the left ventricular free wall from 9 SCD victims with DM and HFpEF and 10 previously healthy individuals who died as a result of an accident were obtained from a Finnish SCD registry as paraffin-embedded sections and analyzed for the expression of seven proteins by immunohistochemistry (see Table 1).

**Table 1:** Study population information.

**No.**	**Gender**	**Age (Years)**	**Hypertension**	**Hypercholesterolemia**	**BMI (kg/m^**2**^)**	**DM status**
SCD victim 1	Male	48	Yes	No	37.6	Type 2
SCD victim 2	Male	70	Yes	No	40.3	Type 2
SCD victim 3	Male	75	No	No	30.8	Type 2
SCD victim 4	Female	52	No	No	76.8	Type 2
SCD victim 5	Male	69	No	Yes	28.4	Type 2
SCD victim 6	Male	67	No	No	31.7	Type 2
SCD victim 7	Male	64	Yes	No	31.2	Type 2
SCD victim 8	Male	63	Yes	No	27.4	Type 2
SCD victim 9	Male	73	Yes	No	42.8	Type 2
Accidental death control 1	Male	70	No	No	22.8	No
Accidental death control 2	Male	46	No	No	26.1	No
Accidental death control 3	Male	49	No	No	32	No
Accidental death control 4	Male	71	Yes	No	41.8	No
Accidental death control 5	Male	76	No	No	28.3	No
Accidental death control 6	Male	64	No	No	21.6	No
Accidental death control 7	Male	67	No	No	34.2	No
Accidental death control 8	Male	69	Yes	Yes	33.3	No
Accidental death control 9	Male	73	No	No	28.9	No
Accidental death control 10	Male	62	No	No	43.7	Type 1

*BMI, Body Mass Index; SCD, Sudden Cardiac Death; DM, Diabetes Mellitus*.

The study complies with the Declaration of Helsinki and was approved by the Ethics Committee of Northern Ostrobothnia Hospital District and the National Authority for Medicolegal Affairs (Valvira). Consent from next of kin was waived by the Ethics Committee since according to the Finnish law, medicolegal autopsy does not require consent.

### Immunohistochemistry

The slides with paraffin-embedded heart sections were incubated for 45 min at 70 degrees, then dewaxed with two 5 min xylene washes and hydrated by 3 min graded ethanol washes of 100% (twice), 95, 80, and 70%, followed by two 4 min water immersions. Heat-mediated antigen retrieval was then done using 1X Citrate Antigen Retrieval Buffer (Ab93678) in a steamer for 75 min followed by permeabilization using 0.2% Triton X-100 (Sigma-Aldrich) in PBS for 30 min, and then blocking with 10% donkey serum in 1% TBST for 45 min. The slides were then incubated with the following antibodies for 2 h at room temperature: osteopontin (hOPN, R&D AF1433; dilution 1:50), low density lipoprotein receptor (LDLR, Abcam Ab52818; dilution 1:200), dynamin 2 (DNM2, Abcam Ab3475; dilution 1:200), fibronectin 1 (FN1, Sigma F3648, dilution 1:200), 2-oxoglutarate dehydrogenase-like (OGDHL, Proteintech 17110-1-AP; dilution 1:200), very low density lipoprotein receptor (VLDLR, Abcam Ab203271; dilution 1:200), and desmoplakin (DSP, Santa Cruz Biotechnology sc-390975, dilution 1:100). Slides were then washed with PBS and staining was detected by incubation with various species-specific biotinylated secondary antibodies [biotinylated goat IgG (Vector Laboratories BA-9500; dilution 1:200), biotinylated rabbit IgG (Vector Laboratories BA-1000, dilution 1:200), biotinylated mouse IgG BA-9200 (Vector Laboratories, dilution 1:200)] at room temperature for 30 min, followed by DAB peroxidase (HRP) amplification (SK-4100, Vector Laboratories). Primary and secondary antibody incubations were done in 10% donkey serum in 1% Tris-Buffered Saline, 0.1% Tween® 20 Detergent (TBST). Each single-antibody staining was repeated 3 times (on 3 different slides from the same paraffin block). No-primary control staining was performed with each of the secondary antibodies used.

### Imaging and Protein Expression Quantification

The stained slides were scanned at 20-X magnification using the Olympus VS120–L100 Virtual Slide Microscope (Tokyo, Japan). Images were quantified using ImageJ (NIH) by 2 independent examiners blinded to the groups. For each sample, 5 images (of 5 fields) were used for quantification and the average positive area normalized to the total stained area (as percentage) was used for statistical analysis.

### Statistics

For all experiments, N refers to the number of individual subjects. All data are expressed as mean ± SEM. *P*-values were calculated using unpaired Student's *t*-tests and *p* < 0.05 was considered significant. Repeated symbols represent *p*-values of different orders of magnitude, i.e., ^*^p < 0.05, ^**^p < 0.01, ^***^p < 0.001, ^****^p < 0.0001. Similar significant differences were confirmed by two independent examiners.

## Results

### Study Population

Table 1 summarizes demographic and clinical information of the SCD victims and accidental death controls. All SCD victims had cardiac cause of death (either ischemic or non-ischemic) with significant coronary artery disease or left ventricular hypertrophy with fibrosis at autopsy.

### Osteopontin and LDLR Are Upregulated in Cardiac Autopsies of SCD Victims With DM+HFpEF

**Figure 1:**
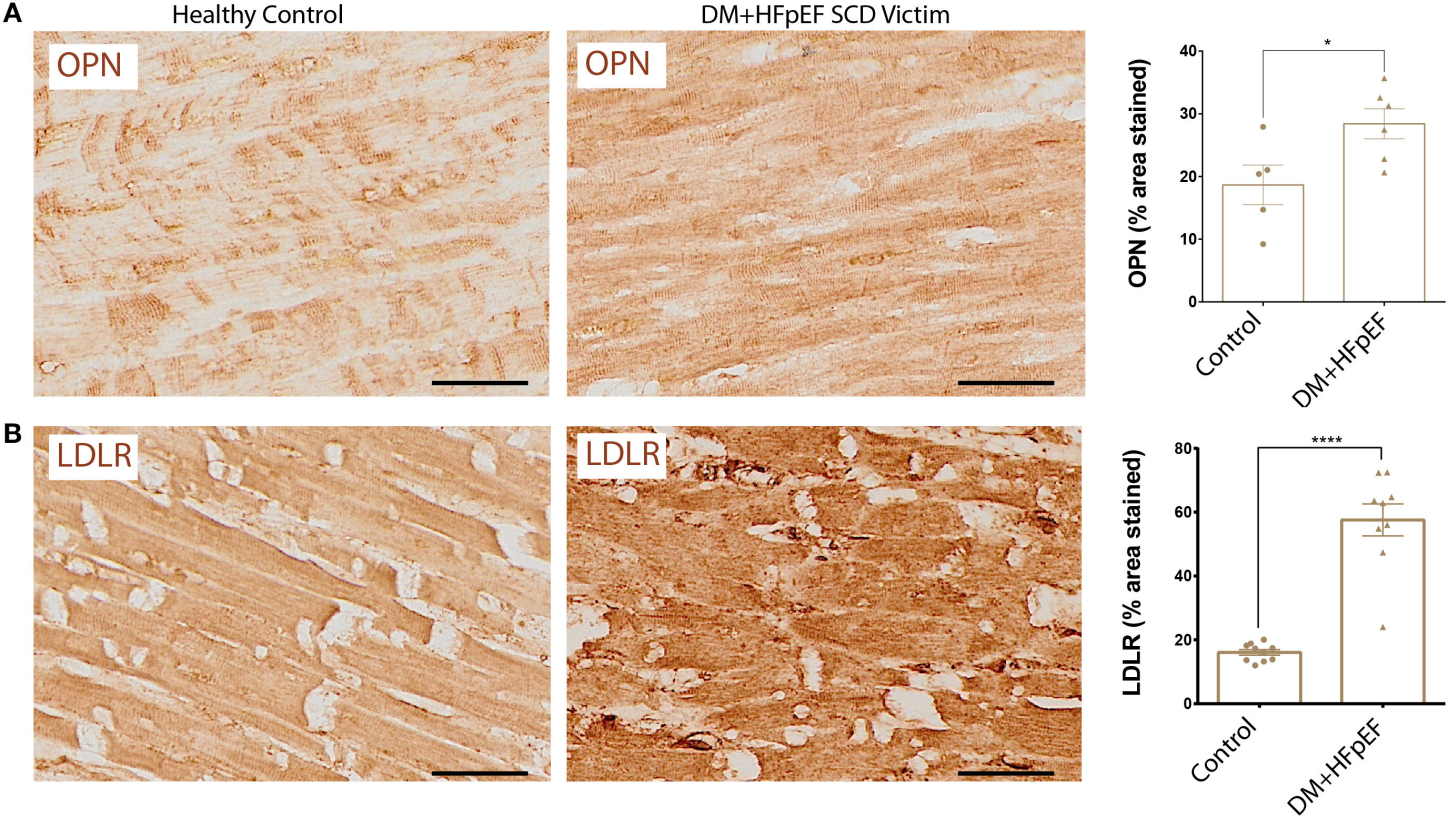
Upregulation of OPN and LDLR in SCD victims with DM+HFpEF. Immunostaining of cardiac biopsies of SCD victims with both HFpEF and DM shows upregulated OPN **(A)** and LDLR **(B)** protein expression compared to control hearts. Data are mean ± SEM. *N* = 5–10 subjects/group. Scale bar = 50 μm. HFpEF, Heart Failure with Preserved Ejection Fraction; DM, Diabetes Mellitus; LDLR, Low Density Lipoprotein Receptor; OPN, Osteopontin. *P < 0.05, ****P < 0.0001 using Student's *t*-test.

We have previously shown that OPN and LDLR are highly expressed in the renal tubules of a mouse model of HFpEF, and that OPN knockcout reduced the pathologically high levels of LDLR and improved renal and cardiac function (8, 14). We found that cardiac OPN expression in hearts of SCD victims with DM+HFpEF was significantly increased by 1.52-fold (28.4 ± 2.4 vs. 18.7 ± 3.2% in the control group, *p* < 0.05, Figure 1A). Moreover, our results showed a 3.58-fold increase in cardiac LDLR protein levels in SCD victims with DM+HFpEF (57.6 ± 5.0 vs. 16.1 ± 0.9% in control group, *p* < 0.0001, Figure 1B).

### DNM2 Is Downregulated in Cardiac Autopsies of SCD Victims DM+HFpEF

**Figure 2:**
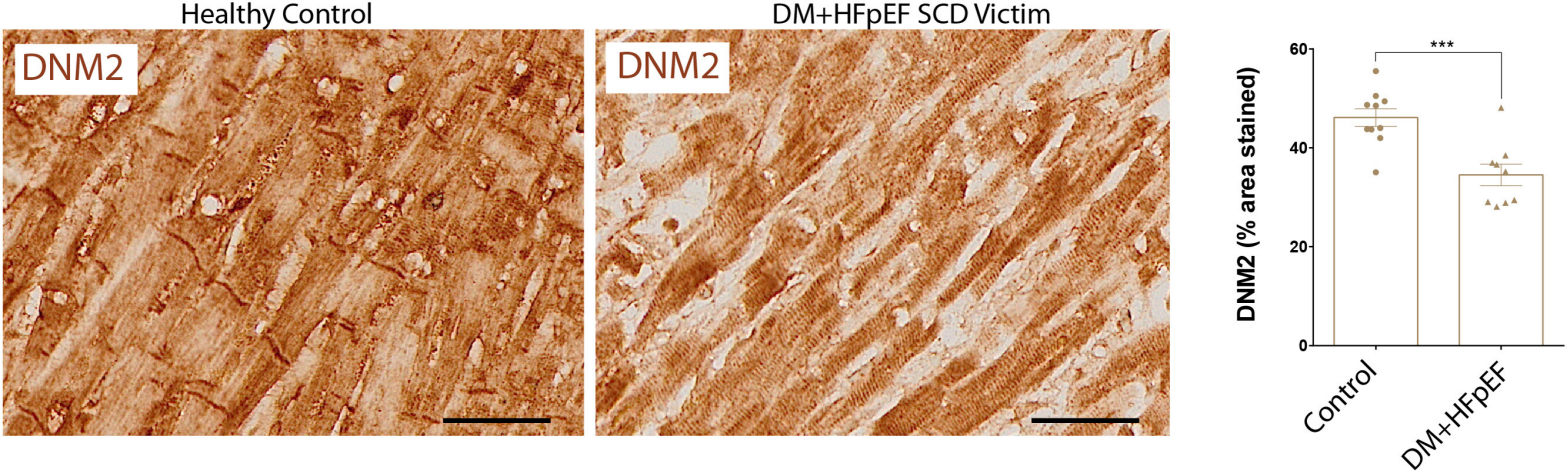
Downregulation of DNM2 in SCD victims with DM+HFpEF. Immunostaining of cardiac biopsies of SCD victims with both HFpEF and DM shows lower DNM2 protein expression compared to control hearts. Data are mean ± SEM. *N* = 9–10 subjects/group. Scale bar = 50 μm. HFpEF, Heart Failure with Preserved Ejection Fraction; DM, Diabetes Mellitus; DNM2, Dynamin-2. ***p < 0.001 using Student's *t*-test.

We have previously shown that OPN and DNM2 are highly expressed in the renal tubules of a mouse model of HFpEF, and that OPN knockcout reduced the pathologically high levels of DNM2 and improved renal and cardiac function (8, 14). When compared with control hearts, cardiac DNM2 expression was significantly lower by 0.69-fold in cardiac tissue from SCD victims with DM+HFpEF (32.3 ± 5.1 vs. 46.5 ± 3.6% in control group, *p* < 0.05, Figure 2).

### FN1 Is Upregulated in Cardiac Autopsies of SCD Victims With DM+HFpEF

**Figure 3:**
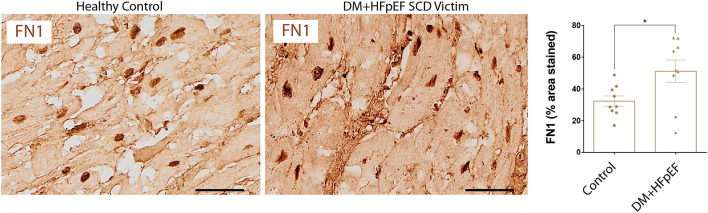
Upregulation of FN1 protein in SCD victims with DM+HFpEF. Immunostaining of cardiac biopsies of SCD victims with both HFpEF and DM shows higher FN1 protein expression compared to control hearts. Data are mean ± SEM. *N* = 9 subjects/group. Scale bar = 50 μm. HFpEF, Heart Failure with Preserved Ejection Fraction; DM, Diabetes Mellitus; FN1, Fibronectin 1. *P < 0.05 using Student's *t*-test.

We have previously shown that in a mouse model of pressure overload, treatment with an OPN aptamer prevented cardiomyocyte hypertrophy and cardiac fibrosis, blocked OPN downstream signaling, and reduced expression of extracellular matrix proteins including FN1 (10). Cardiac FN1 expression in SCD victims with DM+HFpEF was significantly increased by 1.6-fold (51.2 ± 7.1 vs. 32.3 ± 3.3% in control group, *p* < 0.05, Figure 3).

### OGDHL, DSP, and VLDLR Remain Unchanged in Cardiac Autopsies of SCD Victims With DM+HFpEF

**Figure 4:**
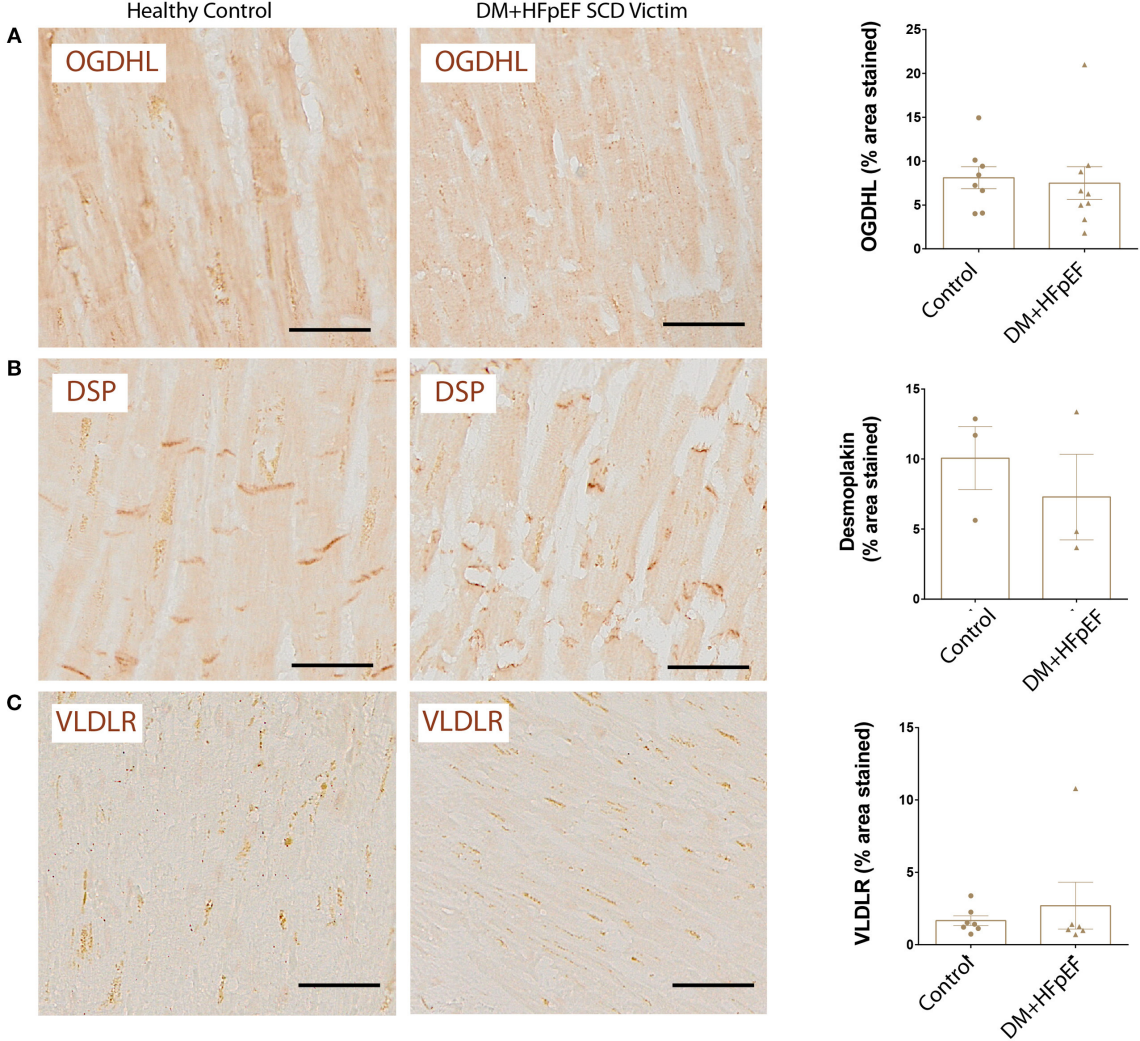
Unchanged expression of OGDHL, DSP, and VLDLR proteins in SCD victims with DM+HFpEF. Immunostaining of cardiac biopsies of SCD victims with both HFpEF and DM shows no significant changes in the expression pattern of OGDHL **(A)**, DSP **(B)** and VLDLR **(C)** compared to control hearts. Data are mean ± SEM. *N* = 3–10 subjects/group. Scale bar = 50 μm. HFpEF, Heart Failure with Preserved Ejection Fraction; DM, Diabetes Mellitus; DSP, Desmoplakin; OGDHL, 2-Oxoglutarate Dehydrogenase Like; VLDLR, Very Low Density Lipoprotein Receptor. No significance was detected by Student's *t*-test.

**Figure 5:**
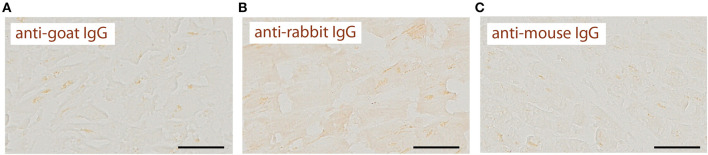
Negative control staining shows no signal. Immunostaining of cardiac biopsies of SCD victims without primary antibodies shows no signal with anti-goat **(A)**, anti-rabbit **(B)** or anti-mouse **(C)** IgG secondary antibodies. Scale bar = 50 μm. These images serve as negative controls for images in Figures 1–4.

We recently reported that OPN negatively regulates the expression of mitochondrial enzyme, OGDHL in the heart of a mouse model of HFpEF, and that OGDHL expression is downregulated in cardiac biopsies from patients with HFpEF and DM (8). We investigated the expression levels of OGDHL, as well as 2 other proteins not previously correlated (DSP and VLDLR) to serve as negative controls, in cardiac tissue from autopsies of SCD victims with HFpEF and DM by immunohistochemistry. Our findings showed that there were no significant changes in the expression patterns of these three proteins (Figure 4). Negative control staining (without primary antibodies) showed no signal as shown in Figure 5.

## Discussion and Conclusions

Diabetes Mellitus (DM) is a major public health problem affecting ~29.1 million Americans. DM is present in about 45% of patients with heart failure with preserved ejection fraction (HFpEF). Furthermore, SCD occurs in ~20% of HFpEF patients (17). Notably, the I-Preserve trial identified DM as a multivariable predictor of SCD in HFpEF patients (18, 19). In HFpEF, type 2 DM comorbidity is associated with smaller left ventricular volumes, worse diastolic dysfunction, poorer quality of life, and overall worse outcomes (20). Among the factors that contribute to increased SCD risk in patients with DM are silent myocardial ischemia, autonomic nervous system dysfunction, and abnormal cardiac repolarization (21). Two important factors that have contributed to current risk stratification in SCD victims who had HFpEF are male gender and insulin-treated DM. In a 2020 study by Adabag and Langsetmo (22), DM was identified as one of the main risk factors associated with significantly increased SCD incidence. In a cohort of 615 HFpEF patients used to validate the survival prediction model, DM was a comorbidity in 52% of SCD victims, while only 29% of the survivors and 36% of those who died due to non-SCD reasons had DM. These results validated the same group's 2014 study (19). which reported that DM was present in 38% of HFpEF patients who died of SCD compared to 35% prevalence in non-SCD deaths and 25% in survivors over a 5 year period. These reports consistently corroborate the elevated risk of SCD in HFpEF patients with DM. The currently known factors that contribute to risk stratification are not useful in lowering mortality attributable to SCD in patients with both DM and HFpEF (17). Thus, the focus of this study was to analyze the expression levels of specific proteins expressed in patients with the phenotype of interest—DM+HFpEF with SCD.

OPN is a matricellular protein that has been implicated in many inflammatory and profibrotic events, especially in the heart. In patients with severe aortic stenosis, elevated OPN levels correlated with increased rates of atrial arrhythmia, and an increased risk of death (23). In our previous work (8, 14), we showed that OPN is highly expressed in kidneys and circulation in a cardio-renal mouse model of HFpEF, and that genetic knock down of OPN reduced HFpEF pathology. The work also showed that OPN regulates LDLR expression *in vivo*. Therefore, we analyzed the protein expression levels of both OPN and its related molecules (previously shown to be correlated to OPN expression), as well as other proteins not previously correlated to OPN, to serve as negative controls.

The results of this study showed that the expression levels of OPN and correlated proteins LDLR, FN1, and DNM2 are highly dysregulated in SCD victims with DM+HFpEF, while those proteins not previously correlated to OPN including VLDLR and DSP were not changed. LDLR, FN1, and OPN showed a significantly increased expression in the diseased hearts, while DNM2 was reduced. Osteopontin had increased expression in the diseased hearts, supporting its role in the pathological process in patients with DM+HFpEF. Increased plasma OPN levels have been directly associated with cardiac dysfunction in HFpEF patients (13). On the other hand, we recently showed that OPN dysregulates the signaling of the β_2_-adrenergic receptor in cardiac cells (7), a receptor known to be associated with SCD in humans (24). Further investigation is required to elucidate the role of elevated OPN levels in SCD victims hearts in regards to SCD pathophysiology.

We had previously demonstrated that OPN regulates LDLR expression and subsequent lipid accumulation and metabolic abnormalities in renal tubules of a mouse model of renal-induced HFpEF (14). Therefore, the elevated LDLR levels in SCD victims with HFpEF and DM could be attributed to the increased OPN levels and may indicate impaired lipid homeostasis in the population studied. FN1 is a matricellular protein that is elevated following myocardial injury or fibrosis (25). Increased expression of FN1 in the diseased hearts alludes to its role in fibrosis that is a major pathology of both DM and HFpEF. DNM2 is involved in endocytosis including LDLR-mediated LDL uptake via clathrin-coated vesicles (26). Therefore, the reduced DNM2 expression in SCD victims could potentially serve a compensatory mechanism to prevent excessive lipid accumulation as a result of LDLR overexpression.

OGDHL is a mitochondrial protein which catalyzes the conversion of 2-oxoglutarate to succinyl-CoA during the TCA cycle and its decreased expression could be associated with mitochondrial dysfunction; impaired energy metabolism could affect efficient glucose utilization (e.g., increased glycolysis and reduced oxidative phosphorylation) (8, 27). In diabetes, OGDHL may have greater consequences for cardiac cells/tissues since there is already reduced TCA cycle flux (28). In our previous study, we detected significant (though modest) changes in OGDHL RNA and protein expression in biopsies obtained from the right ventricular septum from patients with DM and HFpEF (8). However, in this study, we did not detect changes in OGDHL expression by staining in this population.

Our team previously reported that the DSP gene, which codes for the structural protein Desmoplakin, harbors 2 variants associated with sudden cardiac death in subjects with primary myocardial fibrosis (6). Finding no changes in protein expression in another population is not surprising or even expected. In addition, the LDLR and VLDLR are structurally related members of the LDL receptor family with different tissue distribution and functional roles. While the LDLR is predominantly involved in lipid metabolism, the VLDLR has a more limited role in uptake of TG-rich lipoproteins in peripheral tissues [e.g., muscle, heart, adipose tissues (29)]. Compared to the LDLR, the VLDLR binds a variety of non-lipoprotein receptors (thrombospondin-1, LPL, urokinase plasminogen activator /plasminogen activator inhibitor-1 complex, Reelin and fibrin as wells as common ligands with LDLR [LDL, apoE, Lp(a), PCSK9]. These receptors are also differentially regulated; for example, the VLDLR is upregulated by fenofibrate through PPAR-α (30), while the LDLR is not (31). Conversely, the LDLR is downregulated by sterol negative feedback, but the VLDLR is not affected by sterols (32). VLDLR was chosen as a protein that was never correlated with Osteopontin. We found low expression levels of cardiac VLDLR that was not changed between the groups.

**Figure 6:**
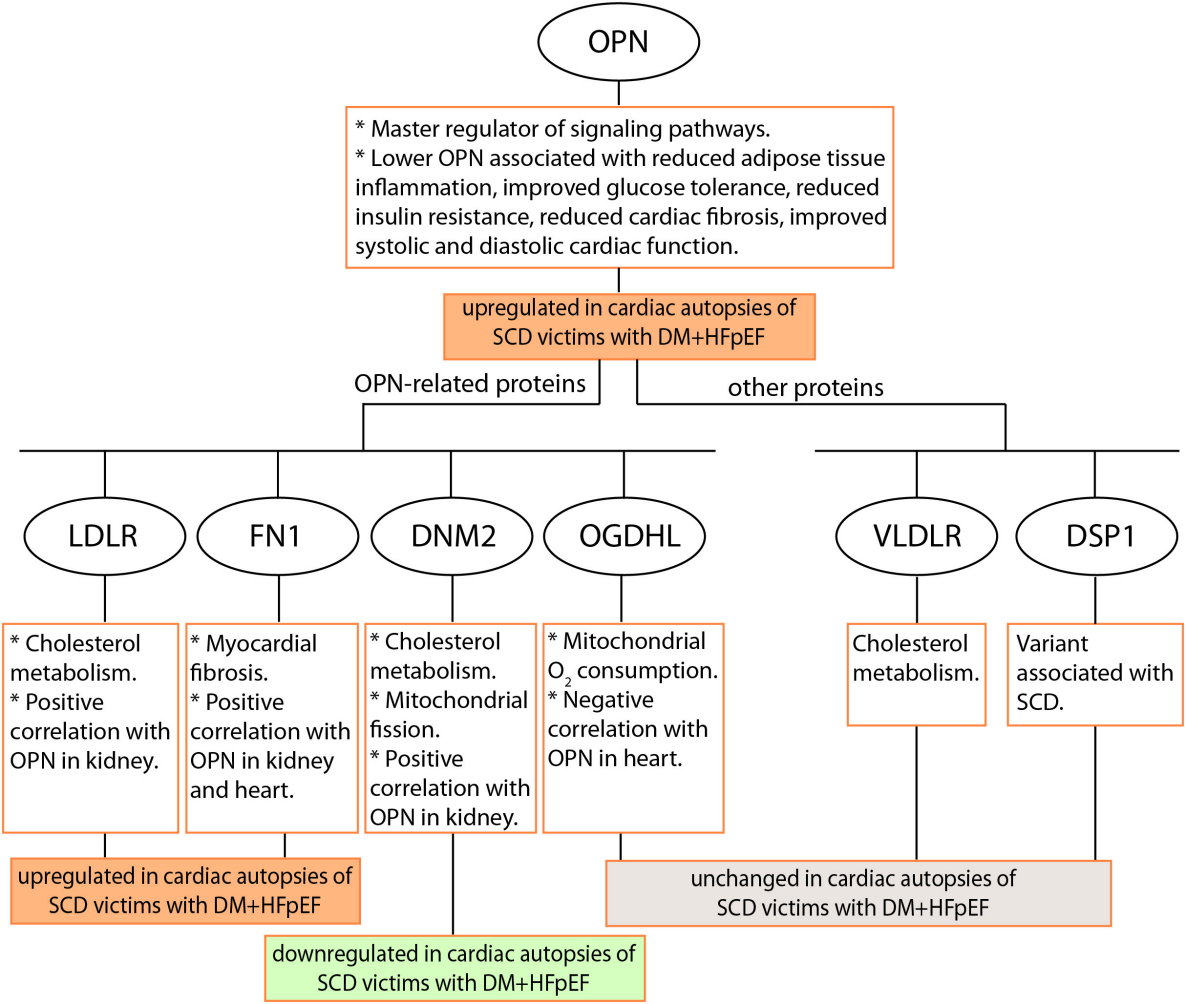
Schematic of the study. Seven proteins were investigated by immunostaining in cardiac autopsies from sudden cardiac death (SCD) victims with Heart Failure with preserved Ejection Fraction (HFpEF) and Type 2 Diabetes Mellitus (DM). OPN, Osteopontin; LDLR, Low Density Lipoprotein Receptor; DNM2, Dynamin-2; FN1, Fibronectin 1; OGDHL, 2-Oxoglutarate Dehydrogenase Like; VLDLR, Very Low Density Lipoprotein Receptor; DSP, Desmoplakin.

The dysregulation of OPN, LDLR, DNM2, and FN1 protein levels in heart tissues (as shown in study schematic in Figure 6) provides supportive evidence and calls for further investigation to elucidate the role(s) and contribution(s) of these potentially detrimental alterations in the pathogenesis of SCD in patients with HFpEF and DM. The fact that secreted OPN and soluble LDLR can be detected in the plasma calls for future investigation of plasma OPN and LDLR levels as risk markers for SCD in patients with DM+HFpEF. Specifically the roles of OPN and LDLR in increased cholesterol accumulation and subsequent pathological effects in the hearts of patients with HFpEF and DM are yet to be elucidated.

## Limitations

This study is limited by the lack of control groups of patients with DM alone, HFpEF alone, and DM+HFpEF without SCD, and by its small numbers. We acknowledge that the levels of LDL-cholesterol, medications history, and HbA1c% would have been informative in our study. Unfortunately, since our subjects are autopsied sudden cardiac death victims, we do not have the detailed blood chemistry and medication history data for all of them. In addition, since the controls are age- and gender- matched accident victims chosen from the Finland registry, we also lack any blood chemistry and medication history data for them.

## Data Availability Statement

The raw data supporting the conclusions of this article will be made available by the authors, without undue reservation.

## Ethics Statement

The studies involving human participants were reviewed and approved by Ethics Committee of Northern Ostrobothnia Hospital District and the National Authority for Medicolegal Affairs (Valvira). The patients/participants provided their written informed consent to participate in this study.

## Author Contributions

KJ-W performed the experiments. MP, DR, KY, and LS analyzed the data and prepared the figures. MP, DR, KY, AL, AM, RG, LT, JG, RM, JJ, and LS interpreted the results of experiments. MP, DR, and KY drafted the manuscript. MP, DR, KY, KJ-W, AL, AM, RG, LT, JG, RM, JJ, and LS edited and revised the manuscript and approved the final version of the manuscript. All authors contributed to the article and approved the submitted version.

## Conflict of Interest

The authors declare that the research was conducted in the absence of any commercial or financial relationships that could be construed as a potential conflict of interest.
